# Personal

**Published:** 1908-10

**Authors:** 


### PERSONAL.

---

Dr. Carl Tompkins, formerly of Randolph, N. Y., has established his office and residence at 106 Elmwood Avenue, Buffalo. Bell telephone Tupper 1511, Frontier 408. Hours: 8 to 9 A.M.; 1 to 3 and 6 P.M. Dr. Tompkins also announces that he is prepared to do clinical laboratory work at his office.

---

Dr. Max Breuer, of Buffalo, who has been hunting in New Brunswick for several weeks, returned the first of October, and has resumed his professional practice at 33 Allen Street.

---

Dr. Dewitt G. Wilcox, of Buffalo, made the principal address at the formal opening of the new surgical building of the Memorial Hospital at Niagara Falls, Thursday evening, September 17, 1908. Four nurses were at the same time graduated at the Training School attached to the institution.

